# Epidemiological features of influenza in Canadian adult intensive care unit patients

**DOI:** 10.1017/S0950268815002113

**Published:** 2015-09-18

**Authors:** G. TAYLOR, K. ABDESSELAM, L. PELUDE, R. FERNANDES, R. MITCHELL, A. McGEER, C. FRENETTE, K. N. SUH, A. WONG, K. KATZ, K. WILKINSON, T. MERSEREAU, D. GRAVEL, the Canadian Nosocomial Infection Surveillance Program (CNISP)

**Affiliations:** 1 University of Alberta Hospital, Edmonton, Alberta, Canada; 2 Public Health Agency of Canada, Centre for Communicable Diseases and Infection Control, Ottawa, Ontario, Canada; 3 Mount Sinai Hospital, Toronto, Ontario, Canada; 4 McGill University Health Centre, Montreal, Quebec, Canada; 5 Ottawa Hospital, Ottawa, Ontario, Canada; 6 Royal University Hospital, Saskatoon, Saskatchewan, Canada; 7 North York General Hospital, Ontario, Canada

**Keywords:** Critical care, epidemiology, hospital, influenza

## Abstract

To identify predictive factors and mortality of patients with influenza admitted to intensive care units (ICU) we carried out a prospective cohort study of patients hospitalized with laboratory-confirmed influenza in adult ICUs in a network of Canadian hospitals between 2006 and 2012. There were 626 influenza-positive patients admitted to ICUs over the six influenza seasons, representing 17·9% of hospitalized influenza patients, 3·1/10 000 hospital admissions. Variability occurred in admission rate and proportion of hospital influenza patients who were admitted to ICUs (proportion range by year: 11·7–29·4%; 21·3% in the 2009–2010 pandemic). In logistic regression models ICU patients were younger during the pandemic and post-pandemic period, and more likely to be obese than hospital non-ICU patients. Influenza B accounted for 14·2% of all ICU cases and had a similar ICU admission rate as influenza A. Influenza-related mortality was 17·8% in ICU patients compared to 2·0% in non-ICU patients.

## INTRODUCTION

Influenza infections occur each autumn and winter season in temperate climates, causing a range of respiratory tract symptom severity from virtually asymptomatic to critical illness. Intensive care units (ICUs) provide supportive care for the most severely affected influenza patients [1, 2]. There have been several epidemiological studies of influenza in ICU patients particularly during the 2009–2010 pandemic but few have evaluated the impact of influenza over multiple seasons [3–10]. Given the year-to-year variability in frequency and severity of influenza infection, a better epidemiological assessment of influenza in ICUs is needed in order to assist clinicians to anticipate the variability and policy-makers to prepare for ICU capacity. The purpose of this report is to examine the frequency, variability, risk factors and outcome of ICU admissions in influenza patients and compare the pandemic year with pre- and post-pandemic periods. We have assessed influenza in hospitalized adults in a network of Canadian hospitals over a 6-year period.

## METHODS

### Surveillance network

The Canadian Nosocomial Infection Surveillance Program (CNISP) is a network of 54 acute-care hospitals from ten Canadian provinces [11, 12]. It is a partnership between the Public Health Agency of Canada (PHAC) and a group of hospital-based Infection Prevention and Control (IPC) specialists, the Canadian Hospital Epidemiology Committee (CHEC) who carry out surveillance for healthcare-associated infections within their facilities. As part of the response to the 2002–2003 SARS pandemic PHAC asked CNISP to establish a respiratory tract infection surveillance programme in network hospitals. CNISP began surveillance for influenza in adults admitted to network hospitals in 2006, as previously described [11, 13]. Clinical practice guidelines recommend testing for influenza in adults admitted to hospital with fever and respiratory symptoms, including community-acquired pneumonia [14]. Surveillance for influenza in adults in participating hospitals is considered to be within the mandate of hospital infection prevention and control programmes and therefore does not constitute human research.

### Surveillance period

From 2006 to 2008, CNISP conducted surveillance of laboratory-confirmed influenza in hospitalized inpatients aged ⩾16 years during the traditional influenza season (November–June). Following the emergence of the pH1N1 influenza virus in 2009, the programme was expanded to year-round surveillance, which continued into the 2010–2011 season. During the 2011–2012 season, the surveillance period returned to the traditional influenza season (November–June).

### Case definition

A case patient was defined as any inpatient aged ⩾16 years with a positive influenza laboratory test result from a specimen collected during the surveillance period who was admitted to an ICU in a participating hospital. Testing by virus culture, antigen detection or nucleic acid testing was performed in accredited laboratories according to national standards [15].

### Case finding

Cases were identified by trained infection control practitioners who identified hospitalized influenza patients at the point of initial positive test. Detailed standardized patient questionnaires were completed for each case and included patients’ laboratory information, patients’ demographic characteristics, and influenza risk factors and comorbidities. Risk factor and comorbidity data were defined on the basis of diagnoses present within the medical record. Patients were reviewed at 30 days following initial positive test to determine antiviral treatment, whether ICU admission had occurred, and clinical outcome. Records of patients who died within 30 days of their initial positive test result were reviewed by a participating physician to determine whether the death was directly related, indirectly related or unrelated to influenza. Data for underlying medical conditions were not available for the 2008–2009 season. Community *vs.* hospital acquisition of influenza was determined by standard definition [11].

### Data analysis

ICU admission rates were calculated using cases who were admitted to an ICU in a participating hospital divided by the number of patient admissions (influenza and non-influenza) in participating hospitals during the surveillance period. To assess admission trends, three time periods were constructed: pre-pandemic (2006–March 2009), pandemic (April 2009–July 2010) and post-pandemic (August 2010–2012).

Age was stratified in two groups, ⩽65 years and >65 years. For each of the three periods, separate contingency tables between ICU admission and each of the patient's and clinical characteristic variables were tested by Fisher's two-sided exact test at the 5% significance level; Wilcoxon's rank sum test was used for continuous variables. The Cochran–Armitage test was used to assess the significance of trend for admission rates in the three different periods. To examine the relationship between ICU admission and patient's and clinical variables simple and multivariable logistic regression models were fit for the pandemic and post-pandemic periods. Final adjusted multivariable logistic regression models were selected by stepwise backward elimination using a cut-off of 0·35 in order to include biologically plausible predictors which otherwise would have been excluded if a lower cut-off was used. There was no multivariable model for the pre-pandemic period due to insufficient data in ICUs. A univariate logistic regression model was fit to assess death due to influenza in ICU patients with each of the possible patient's and clinical predictors. Individuals with missing data in any of the logistic regression models were censored in the analysis. All statistical analyses were performed using SAS Enterprise Guide 5·1 (SAS Institute Inc., USA).

## RESULTS

**Fig. 1.**
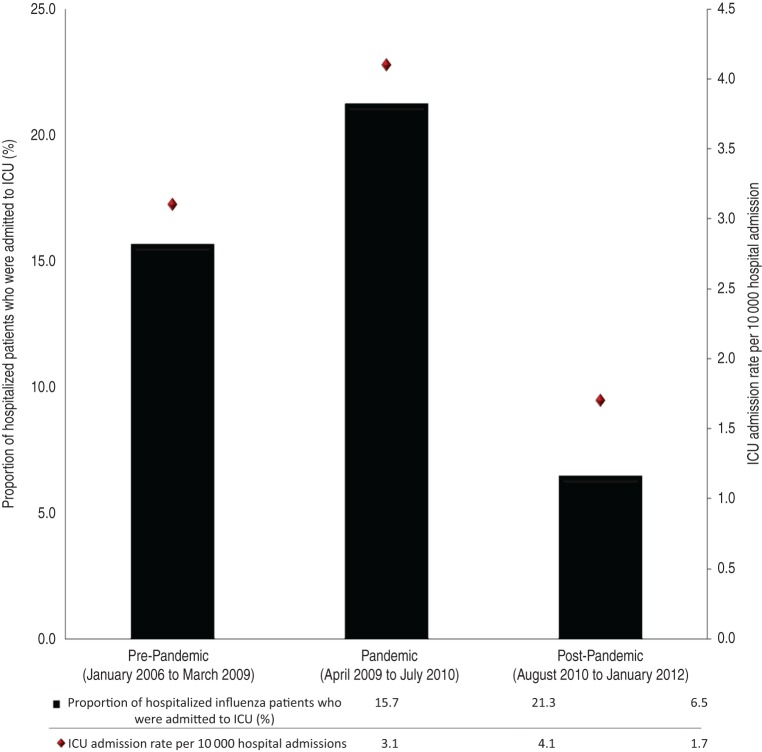
ICU influenza admission rates and proportions by pre-pandemic, pandemic and post-pandemic periods. Cochran–Armitage trend test *P* value 0·017.

In the six study years there were 626 adult patients with virologically confirmed influenza admitted to an ICU in a participating hospital, ranging from 97 cases in the pre-pandemic period to 404 in the pandemic year; 78·0% of ICU-admitted patients also required mechanical ventilation. ICU cases represented 17·9% of all patients admitted to hospital with influenza (range by year 11·7% in 2006–2007 to 29·4% in 2008–2009 and from a low of 6·5% in the post-pandemic period to a high of 21·3% in the pandemic period; Fig. 1). Overall ICU admission rate was 3·1/10 000 hospital admissions ranging from 1·7/10 000 in the post-pandemic period to 4·1/10 000 in the pandemic period and by year from 1·7/10 000 (2006–2007) to 4·7/10 000 (2009–2010). There was a significant trend across the three different periods in the ICU admission rate (*P* = 0·01). Influenza B was identified in 89 (14·2%) cases. Table 1 compares characteristics of hospitalized ICU and non-ICU influenza patients based on the three different periods. Univariate analysis revealed no differences between the age groups in ICU and non-ICU patients during the pre-pandemic period. In the pandemic period, ICU patients were younger (median 52, min 16, max 97 years), more likely to be infected with influenza acquired in the community, suffer from cardiac disease and/or obesity. In the post-pandemic period, ICU patients were also younger (median 62, min 18, max 97 years), more likely to be male and suffer from a pulmonary/chronic lung disease. The proportion of patients suffering from a chronic illness in general was similar for ICU and non-ICU groups in both the pre-pandemic and pandemic period but different in post-pandemic. In all periods, ICU patients had a higher proportion of 30-day all-cause mortality (ranging from 20·9% to 27·8% in ICU *vs.* 2·6% to 4·2% in non-ICU, *P* < 0·0001), and were more likely to be treated with antiviral therapy than non-ICU patients (*P* < 0·005 in all three different periods). Vaccination rate for ICU patients was low, ranging from 62·1% (pre-pandemic) to 26·4% (pandemic) (Table 1). Influenza mortality was 17·8% in ICU patients compared to 2·0% in non-ICU patients and did not significantly differ between influenza A and B.

**Table 1.** Characteristics of Canadian ICU and non-ICU hospital influenza patients by pre-pandemic, pandemic and post-pandemic period

	Pre-pandemic period (2006–2009)			Pandemic period (2009–2010)			Post-pandemic period (2010–2012)		
	ICU (%)	Non-ICU (%)		ICU (%)	Non-ICU (%)		ICU (%)	Non-ICU (%)	
Variables	(*N* = 97)	(*N* = 534)	*P* value*	(*N* = 404)	(*N* = 1497)	*P* value*	(*N* = 125)	(*N* = 835)	*P* value*
Age, yr, median±(min, max)	67 (19, 96)	72 (16, 98·11)	0·1495	52 (16, 97)	56 (16, 101)	<0·0001	62 (18, 97)	74 (16, 104)	<0·0001
**Age (category)**			
>65 years	53/97 (54·6)	316/534 (59·2)	0·4338	85/404 (21·0)	560/1497 (37·4)	<0·0001	59/125 (47·2)	527/835 (63·1)	<0·0001
Gender			
Male	50/97 (51·5)	265/532 (49·8)	0·8254	191/404 (47·3)	697/1497 (46·6)	0·8222	69/125 (55·2)	360/835 (43·1)	0·0122
Vaccine status (yes)	18/29 (62·1)	115/220 (52·3)	0·4288	43/163 (26·4)	185/566 (32·7)	0·1502	15/43 (34·8)	157/342 (45·9)	0·1947
**Influenza type**			
Influenza B	25/97 (25·8)	154/534 (28·8)	0·3323	15/404 (3·7)	76/1497 (0·05)	<0·0001	49/125 (39·2)	309/835 (37·0)	0·0885
**Source of influenza**			
Healthcare associated	16/94 (17·0)	127/525 (24·2)	0·1447	35/380 (9·2)	221/1373 (16·1)	<0·0001	16/120 (13·3)	154/800 (19·2)	0·1310
Chronic illness	56/67 (83·6)	392/462 (84·8)	0·8558	321/395 (81·3)	1237/1487 (83·2)	0·3689	117/125 (93·6)	723/835 (86·6)	0·0287
Asthma	0	0	–	24/118 (20·3)	80/686 (11·7)	0·0165	19/125 (15·2)	100/835 (12·0)	0·3091
Diabetes	13/67 (19·4)	76/462 (16·5)	0·5996	66/378 (17·5)	269/1389 (19·4)	0·4167	33/124 (26·6)	181/800 (22·6)	0·3599
Cardiac disease	23/67 (34·3)	91/462 (19·7)	0·0103	66/378 (17·5)	371/1389 (26·7)	<0·0001	32/124 (25·8)	231/800 (28·9)	0·5222
Liver disease*	0	0	–	1/101 (1·0)	16/676 (2·3)	0·4917	8/125 (6·4)	18/835 (2·2)	0·0134
Renal/kidney disease	5/67 (7·5)	38/462 (8·2)	0·9999	35/378 (9·3)	120/1389 (8·6)	0·6828	10/124 (8·1)	68/800 (8·5)	0·9999
Pulmonary/lung disease	22/67 (32·8)	119/462 (25·8)	0·2377	136/378 (36·0)	515/1389 (37·0)	0·7185	53/124 (42·7)	242/800 (30·3)	0·0070
Immunocompromised/cancer	10/67 (14·9)	65/462 (14·1)	0·8517	70/378 (18·5)	251/1389 (18·1)	0·8219	31/124 (25·0)	147/800 (18·4)	0·0872
Obesity	0/2 (0)	1/7 (14·3)	0·9999	41/404 (10·1)	28/1497 (1·9)	<0·0001	3/125 (2·4)	17/835 (2·0)	0·7367
30-day Mortality	32/96 (33·3)	20/534 (3·7)	<0·0001	85/404 (21·0)	39/1497 (2·6)	<0·0001	30/124 (24·2)	35/831 (4·2)	<0·0001
Influenza primary cause	7/29 (24·1)	3/17 (17·6)	0·7264	31/78 (39·7)	8/35 (22·9)	0·0132	3/24 (12·5)	6/32 (18·8)	0·2030
Influenza contributing	8/29 (27·6)	7/17 (41·2)		42/78 (53·9)	18/35 (51·4)		17/24 (70·8)	15/32 (46·9)	
Mechanical ventilation (yes)	64/94 (68·1)	2/532 (0·3)	<0·0001	325/404 (80·4)	6/1497 (0·4)	<0·0001	44/57 (77·2)	1/369 (0·3)	<0·0001
Use of antiviral therapy	52/97 (53·6)	198/517 (38·3)	0·0067	364/392 (92·8)	1235/1478 (83·6)	<0·0001	103/125 (82·4)	557/805 (69·2)	0·0021

ICU, Intensive care unit.

Median age presented and Wilcoxon's rank sum test used to assess differences.

The ICU sample size excludes individuals who were already admitted into ICU prior to becoming infected with influenza (*n* = 45).

* Fisher's two-sided exact test.

Table 2 provides the unadjusted estimates of risk factors for ICU admission. In the pre-pandemic period, no risk factors were found to be significant. In the pandemic period, the older age group had reduced risk of ICU admission with an odds ratio (OR) of 0·41 [95% confidence interval (CI) 0·31–1·54]; whereas community-acquired influenza (OR 2·60, 95% CI 1·69–4·00), and obesity (OR 6·20, 95% CI 3·78–10·16) were associated with higher risk of being admitted to the ICU. Cardiac disease was associated with reduced risk of being admitted to the ICU (OR 0·55, 95% CI 0·41–0·75). In the post-pandemic period, the older age group was also associated with reduced risk of being admitted to the ICU (OR 0·56, 95% CI 0·38–0·82) while male sex (OR 1·54, 95% CI 1·04–2·28) and pulmonary/chronic lung disease (OR 1·87, 95% CI 1·25–2·78) were associated with an increased risk.

**Table 2.** Unadjusted ORs* in influenza infected patients admitted to the ICU† stratified by pre-pandemic, pandemic and post-pandemic periods

	Pre-pandemic period‡ (2006–2009)			Pandemic period (2009–2010)			Post-pandemic period (2010–2012)		
	(*N* = 631)			(*N* = 1901)			(*N* = 960)		
Variables	ICU/total	OR (95% CI)	*P* value	ICU/total	OR (95% CI)	*P* value	ICU/total	OR (95% CI)	*P* value
Age group									
>65 *vs*. ⩽65 years	97/631	1·20 (0·78–1·86)	0·4046	404/1901	0·45 (0·34–0·58)	<0·0001	125/960	0·52 (0·36–0·77)	0·0008
Gender									
Male *vs*. female	97/629	1·0 (0·70–1·65)	0·7534	404/1497	1·03 (0·83–1·28)	0·7975	115/950	1·63 (1·14–2·37)	0·0118
Vaccine status									
Non-vaccinated *vs*. vaccinated	220/249	0·67 (0·30–1·48)	0·3226	566/729	1·35 (0·92–2·00)	0·1269	43/385	0·63 (0·33–1·22)	0·1733
Influenza type									
Influenza A *vs*. influenza B	97/534	1·14 (0·70–1·87)	0·1793	404/1901	0·88 (0·49–1·58)	0·6759	125/960	1·29 (0·85–1·96)	0·2310
Source of influenza									
Community *vs*. healthcare acquired	94/524	1·56 (0·88–2·76)	0·1312	380/1753	1·89 (1·30–2·76)	0·0009	111/911	1·55 (0·89–2·70)	0·1219
Chronic illness (yes *vs*. no)									
Cardiac disease	67/529	2·13 (1·23–3·71)	0·0074	378/1767	0·58 (0·43–0·78)	0·0002	124/924	0·86 (0·56–1·32)	0·4814
Pulmonary/lung disease		1·41 (0·81–2·45)	0·2221		0·95 (0·75–1·21)	0·6958		1·71 (1·17–2·53)	0·0059
Immunoompromised/cancer		1·07 (0·52–2·20)	0·8511		1·03 (0·77–1·38)	0·8404		1·48 (0·95–2·31)	0·0832
Obesity		–	–	404/1901	5·92 (3·61–9·71)	<0·0001	115/950	1·18 (0·34–4·10)	0·7906

ICU, Intensive care unit; OR, odds ratio; CI, confidence interval.

* OR calculated by a univariate logistic regression.

† Includes influenza infected individuals admitted to ICU, excludes patients already admitted to ICU prior to being infected by influenza.

‡ Pre-pandemic did not have any cases of obesity in the ICU group.

For the pandemic period, the logistic regression model was adjusted for age, gender, influenza strain type, source of the pathogen and for pulmonary/chronic lung disease (Table 3). This model identified that age >65 years was associated with reduced risk of ICU admission (OR 0·60, 95% CI 0·43–0·84) while obesity (OR 5·03, 95% CI 2·91–8·69) was associated with increased risk of ICU admission. During the pandemic, the OR for ICU admission of obese patients aged ⩽65 years was 2·00 (95% CI 1·48–9·79).

**Table 3.** Fully adjusted ORs obtained by multivariate logistic regression model via backward elimination procedure of admittance to the ICU in influenza infected patients stratified by pandemic, and post-pandemic period

	Pandemic period* (2009–2010)			Post-pandemic period† (2010–2011)		
	(*N* = 1618)			(*N* = 884)		
Risk factors	ICU (*n*)	OR	95% CI	ICU (*n*)	OR	95% CI
Age >65 years		0·62	(0·45–0·85)		0·54	(0·36–0·80)
Chronic illness						
Obesity	353	4·84	(2·81–8·33)	119	–	–
Pulmonary/lung disease		–	–		1·99	(1·33–2·99)
Immunocompromised/cancer		1·15	(0·84–1·59)		1·31	(0·81–2·10)
Overall		1·99‡	(1·38–2·87)		0·94§	(0·28–1·54)
						

ICU, Intensive care unit; OR, odds ratio; CI, confidence interval.

* Multivariate model was adjusted for age, gender, influenza strain type, H1N1 pandemic strain, source of the pathogen and chronic illness: immunocompromised/cancer and obesity. The corresponding Hosmer–Lemeshow test statistic for model fit (*P* = 0·68).

† Multivariate model was adjusted for age, gender, influenza strain type, source of the pathogen and chronic illness: pulmonary/lung disease and immunocompromised/cancer. The corresponding Hosmer–Lemeshow test statistic for model fit (*P* = 0·86).

‡ Pandemic overall OR reflects if the individual is aged ⩽65 years, obese and was infected with H1N1 pandemic strain.

§ Post-pandemic overall OR reflects if the individual is aged ⩽65 years, and suffers from pulmonary/lung disease.

For the post-pandemic period, a multivariable logistic regression model was adjusted for age, gender, source of the infection and chronic illness including pulmonary/lung disease and immunocompromised/cancer. Once again age >65 years demonstrated a reduced risk for ICU admission (OR 0·57, 95% CI 0·37–0·87), whereas, suffering from a pulmonary/chronic lung disease was associated with an increased risk of ICU admission (OR 2·05, 95% CI 1·35–3·11).

In Table 4, a univariate logistic regression on the 30-day mortality revealed significance only for being admitted during the pandemic period (*P* = 0·001) and borderline significance for being admitted in the post-pandemic period (*P* = 0·0446). The corresponding unadjusted ORs were 13·37 (95% CI 2·53–41·15) and 5·57 (95% CI 1·04–29·79), respectively.

**Table 4.** Unadjusted ORs describing the association between the potential risk factors and the 30-day mortality in ICU-admitted patients (n = 118)*

Risk factors	Proportion	OR (95% CI)	*P* value
Age (category)			
>65 *vs*. ⩽65	77/118	0·76 (0·28–2·05)	0·5888
Gender			
Male *vs*. female	57/118	1·17 (0·45–3·08)	0·7457
Vaccine status			
Non-vaccinated *vs*. vaccinated	26/40	4·8 (0·75–30·55)	0·0967
Source of influenza			
Community *vs*. healthcare acquired	91/108	1·00 (0·25–3·91)	0·9949
Chronic illness (yes *vs*. no)			
Cardiac disease	30/108	0·88 (0·30–2·54)	0·8059
Pulmonary/lung disease (*n* = 49)	49/108	1·31 (0·49–3·51)	0·5937
Immunocompromised/cancer (*n* = 26)	26/108	0·69 (0·23–2·02)	0·4937
⩾1 chronic illness (*n* = 98)	98/110	0·89 (0·18–4·41)	0·8854
Mechanical ventilation (yes *vs*. no)	106/118	1·75 (0·43–7·12)	0·4376
Pandemic period (*vs*. pre-pandemic)	77/108	13·37 (4·11–43·50)	<0·0001
Post-pandemic period (*vs*. pre-pandemic)	14/108	5·57 (1·04–29·79)	0·0446

OR, Odds ratio; CI, confidence interval.

* 30-day mortality due to influenza (*n* = 98) and non-related to influenza (*n* = 20).

## DISCUSSION

We document the large impact of influenza on ICUs, with 17·9% of all hospitalized patients requiring an ICU stay, rising to >25% in some influenza seasons. This is similar to a population-based US study by Reed *et al*. (excluding influenza B cases during the pandemic year) which found that 19·2% of adults hospitalized between 2005 and 2010 were admitted to an ICU [10], although our 78% mechanical ventilation rate for ICU patients appears higher than that study. Our study found that the ICU admission rate increased during the 2009–2010 pandemic but fell below the pre-pandemic rate in the two post-pandemic seasons. Clearly, predicting the impact of influenza on ICU admissions in any given season is extremely difficult and mechanisms to manage a surge of cases should be available for every season.

In our study patients admitted to the ICU were younger than other hospitalized influenza patients, even in the two post-pandemic seasons. Other risk factors for ICU admission were variable. Obesity was the only identified ICU risk factor during the pandemic. Presence of cardiac disease was associated with reduced ICU admission risk during the pandemic but increased risk pre-pandemic. Post-pandemic, chronic pulmonary disease was identified as a risk factor. Previous studies examining ICU admission risk have primarily examined pandemic years, with few studies examining seasonal influenza, as reviewed by Mertz *et al*. [16]. An explanation for our finding of younger age as an ICU admission risk factor is not readily apparent. Plausibly, previous seasonal infections or more frequent seasonal vaccination in older individuals protects against disease severe enough to warrant ICU care [17]. Alternatively, the elderly may be admitted to hospital more frequently with clinically less severe influenza or out of personal choice may be less frequently transferred for ICU care and mechanical ventilation when severe illness is present. Obesity was found to be a risk factor for severe disease during the 2009–2010 pandemic, and has been associated with seasonal influenza hospitalization and mortality but has not previously been found to be an ICU risk factor during seasonal influenza [16, 18–23]. As reviewed by Seema & Chaves, a number of biological mechanisms associating obesity with influenza have been postulated [24]. We did not collect data on pregnancy in ICU patients which has been identified as a risk factor for severe influenza [18, 25].

Despite the high prevalence of comorbidities in ICU patients (>85%) only one third had been vaccinated against influenza; given an average seasonal vaccine efficacy of 59%, there is clearly an opportunity to reduce the ICU burden by increasing population vaccine uptake [4, 8, 26]. There has been controversy as to whether influenza B is of the same or lesser severity compared to influenza A strains [27, 28]. Our logistic regression models found no difference supporting the findings of a recent US study [29]. We did not collect data on influenza subtypes and so cannot say whether this finding applies to all influenza A subtypes.

ICU patients with influenza had a 23·6% all-cause and 17·8% influenza-attributable 30-day mortality, compared to 2% for non-ICU patients, documenting the severe impact of disease in this group despite ICU care. Again, influenza A and B ICU patients had similar mortality. Our study did not identify cardiac disease, immunosuppression or neuromuscular disease as risk factors for mortality, which have been reported as mortality risk factors in other studies [16]. Since our mortality risk factor study examines mortality once a patient has reached an ICU, patients dying before reaching an ICU would not be included.

While this study represents the largest epidemiological survey of influenza in ICU patients to date it does have some limitations. The hospital network represents primarily large urban teaching hospitals; it is likely that patients in these hospitals differ from those in smaller community hospitals. Our study represents observational data; as in any laboratory-based passive surveillance system testing frequency and criteria for ICU admission may change over time. Comorbidity data and vaccination status were inconsistently recorded in the medical records; vaccination status was only recorded for 37·5% (*n* = 235) of ICU patients. Influenza A subtyping was inconsistently performed, limiting our ability to assess variation in risk factors and outcomes of subtypes.

In conclusion the proportion of patients hospitalized with influenza who require ICU care is high, although this proportion and ICU admission rates vary substantially year to year. ICU patients are younger and more likely to be obese but not otherwise distinguishable from non-ICU patients. Vaccination rate in ICU patients is very low; improving vaccine uptake represents an opportunity to reduce burden of disease in patients and ICUs. Influenza B was found to be of similar severity and outcome to influenza A (all types).

## APPENDIX

### Members of The Canadian Nosocomial Infection Surveillance Program

David Boyd, National Microbiology Laboratory, Public Health Agency of Canada; Natalie Bridger, Eastern Health-Health Sciences Centre, St. John's, Newfoundland; Elizabeth Bryce, Vancouver Coastal Health Authority, Vancouver, British Columbia; John Conly, Foothills Medical Centre, Calgary, Alberta; André Dascal, Sir Mortimer B. Davis–Jewish General Hospital, Montreal, Quebec; Janice de Heer, Interior Health Authority, Kelowna, British Columbia; John Embil, Health Sciences Centre, Winnipeg, Manitoba; Joanne Embree, Health Sciences Centre, Winnipeg, Manitoba; Gerald Evans, Kingston General Hospital, Kingston, Ontario; Sarah Forgie, Stollery Children's Hospital, Edmonton, Alberta; Charles Frenette, McGill University Health Centre, Montreal, Quebec; David Haldane, Queen Elizabeth II Health Sciences Centre, Halifax, Nova Scotia; Gregory German, Queen Elizabeth Hospital, Charlottetown, Prince Edward Island; George Golding, National Microbiology Laboratory, Public Health Agency of Canada; Denise Gravel, Centre for Communicable Diseases and Infection Control, Public Health Agency of Canada; Deanna Hembroff, University Hospital of Northern British Columbia, Prince George, British Columbia; Elizabeth Henderson, Alberta Health Services, Calgary, Alberta; Michael John, London Health Sciences Centre, London, Ontario; Lynn Johnston, Queen Elizabeth II Health Sciences Centre, Halifax, Nova Scotia; Kevin Katz, North York General Hospital, Toronto, Ontario; Pamela Kibsey, Victoria General Hospital, Victoria, British Columbia; Magdalena Kuhn, South East Regional Health Authority, Moncton, New Brunswick; Joanne Langley, IWK Health Centre, Halifax, Nova Scotia; Camille Lemieux, University Health Network, Toronto, Ontario; Nicole Le Saux, Children's Hospital of Eastern Ontario, Ottawa, Ontario; Mark Loeb, Hamilton Health Sciences Corporation, Hamilton, Ontario; Susan Richardson, Hospital for Sick Children, Toronto, Ontario; Allison McGeer, Mount Sinai Hospital, Toronto, Ontario; Dominik Mertz, Hamilton Health Sciences Corporation, Hamilton, Ontario; Mark Miller, Sir Mortimer B. Davis-Jewish General Hospital, Montreal, Quebec; Robyn Mitchell, Centre for Communicable Diseases and Infection Control, Public Health Agency of Canada; Dorothy Moore, Montreal Children's Hospital, McGill University Health Centre, Montreal, Quebec; Aboubakar Mounchili, Centre for Communicable Diseases and Infection Control, Public Health Agency of Canada; Michael Mulvey, National Microbiology Laboratory, Public Health Agency of Canada; Suzanne Pelletier, Health Sciences North, Sudbury, Ontario; Linda Pelude, Centre for Communicable Diseases and Infection Control, Public Health Agency of Canada; Caroline Quach, Montreal Children's Hospital, McGill University Health Centre, Montreal, Quebec; Virginia Roth, The Ottawa Hospital, Ottawa, Ontario; Andrew Simor, Sunnybrook Health Sciences Centre, Toronto, Ontario; Stephanie Smith, University of Alberta Hospital, Edmonton, Alberta; Kathryn Suh, The Ottawa Hospital, Ottawa, Ontario; Geoffrey Taylor, University of Alberta Hospital, Edmonton, Alberta; Eva Thomas, Children's and Women's Health Center, Vancouver, British Columbia; Nathalie Turgeon, Centre Hospitalier de Universitaire de Québec–Hôtel-Dieu, Quebec, Quebec; Mary Vearncombe, Sunnybrook Health Sciences Centre, Toronto, Ontario; Joseph Vayalumkal, Alberta Children's Hospital, Calgary, Alberta; Karl Weiss, Maisonneuve-Rosemont Hospital, Montreal, Quebec; Alice Wong, Royal University Hospital, Saskatoon, Saskatchewan.
